# Supplementary material for: Native mass spectrometry and structural studies reveal modulation of MsbA–nucleotide interactions by lipids
Source: Nat Commun. 2024 Jul 15;15:5946. doi: 10.1038/s41467-024-50350-9 (PMC11251056; doi:10.1038/s41467-024-50350-9)
Supplement: Supplementary file 1 — Supplementary Information [file 41467_2024_50350_MOESM1_ESM.pdf]

Supplementary Information

Native mass spectrometry and structural studies reveal modulation of MsbA-nucleotide interactions by lipids

Tianqi Zhang[1], Jixing Lyu[1], Bowei Yang[2], Sangho D. Yun[1], Elena Scott[1], Minglei Zhao[2], Arthur Laganowsky[1,*]

[1]Department of Chemistry, Texas A&M University, College Station, TX 77843
[2]Department of Biochemistry and Molecular biology, University of Chicago, Chicago, IL 60637
*Correspondence to: ALaganowsky@chem.tamu.edu

Supplementary Figures

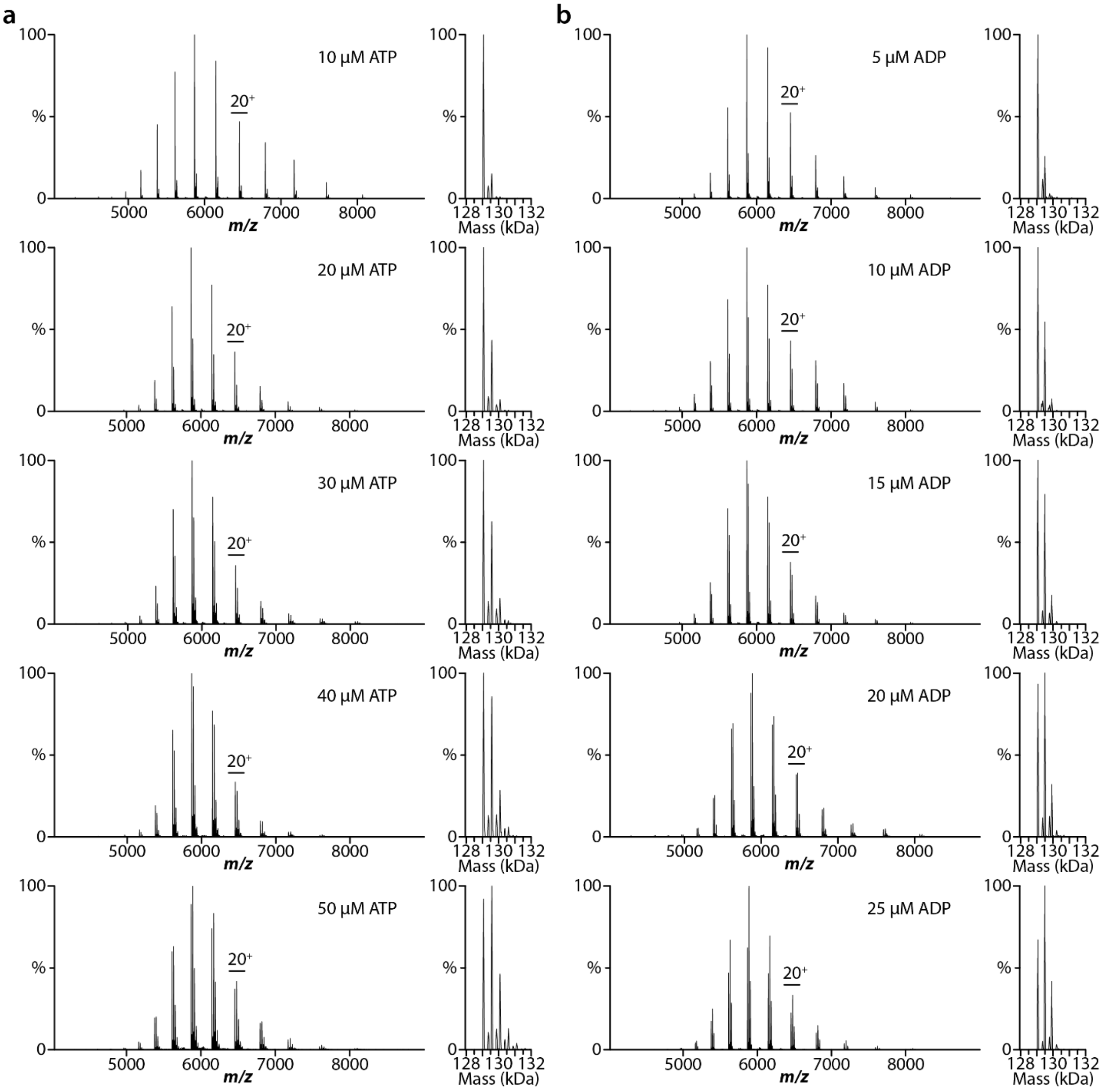

Supplementary Fig. 1. Representative native mass spectra for nucleotide binding to MsbA. MsbA (0.5 µM) was mixed with 10 µM Mg^{2+} and different concentrations of **a** ATP and **b** ADP. Deconvoluted mass spectra are shown to the right.

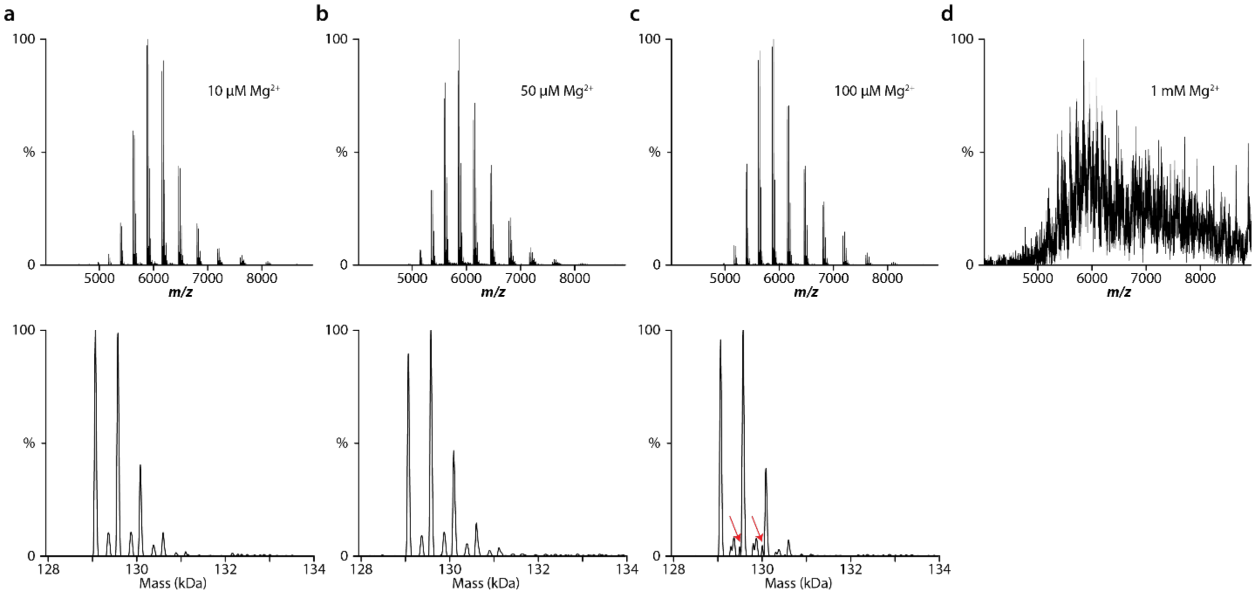

Supplementary Fig. 2. Native mass spectra for nucleotide binding to MsbA in the presence of different concentrations of Mg^{2+}. MsbA (0.5 µM) was mixed with 50 µM ATP and different concentrations of Mg^{2+}. Deconvoluted mass spectra are shown below with peaks corresponding to ADP binding denoted with an arrow.

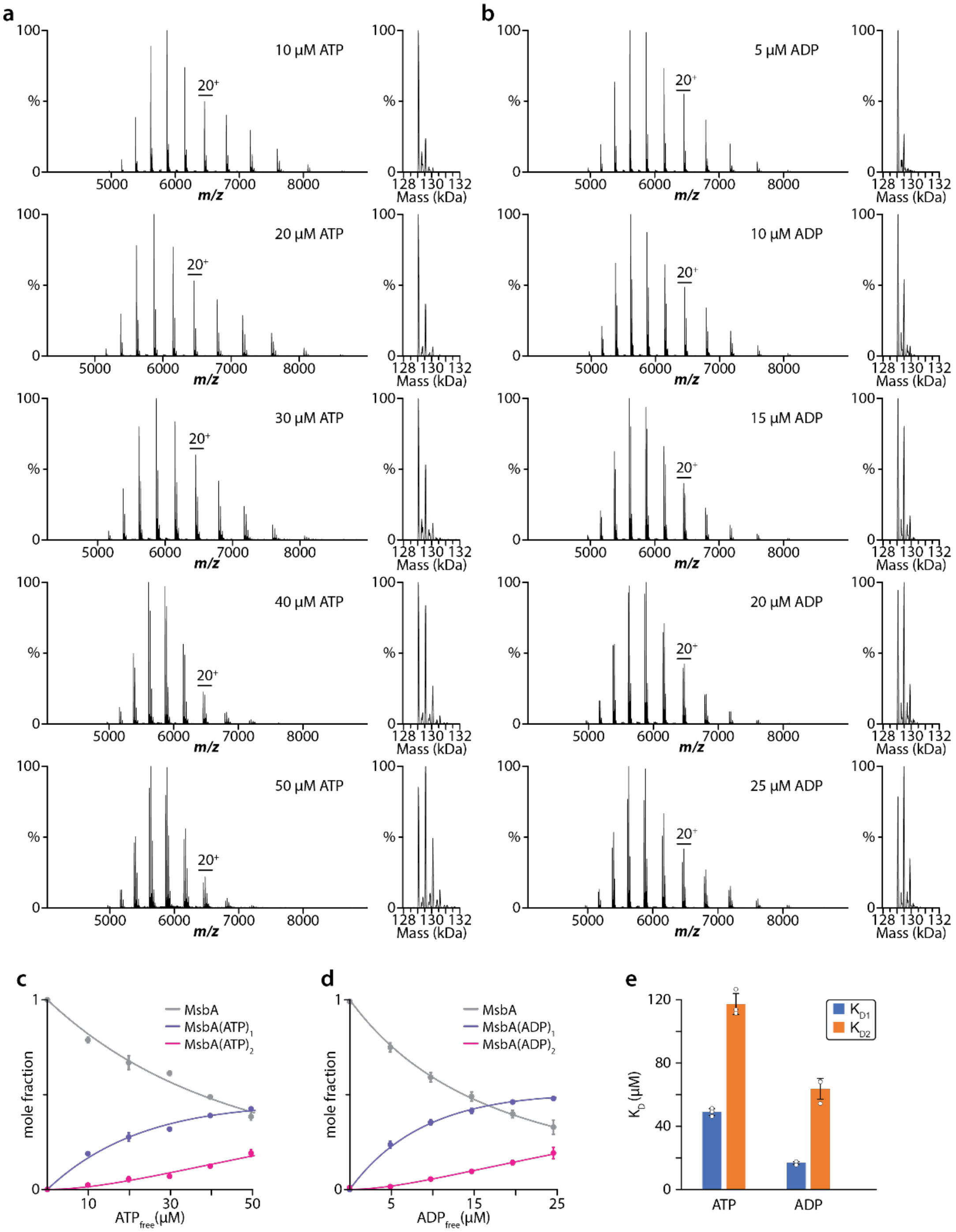

Supplementary Fig. 3. Determination of equilibrium dissociation constants (K_D) for individual nucleotide-binding events to MsbA in the presence of 50 µM Mg^{2+}. Representative native mass spectra and the deconvolution for nucleotide binding to MsbA. MsbA (0.5 µM) was mixed with 50 µM Mg^{2+} and different concentrations of **a** ATP and **b** ADP. **c** Plot of mole fraction data for MsbA(ATP)$_{0\text{-}2}$ determined from the titration series (dots) and resulting fit from a sequential ligand-binding model (solid lines). **d** Plot of mole fraction data for MsbA(ADP)$_{0\text{-}2}$ determined as described for panel c. **e** K_{Dn} values for the nth nucleotide binding to MsbA. Reported are the mean and standard deviation (n = 3, biological replicates). Source data are provided as a Source Data file.

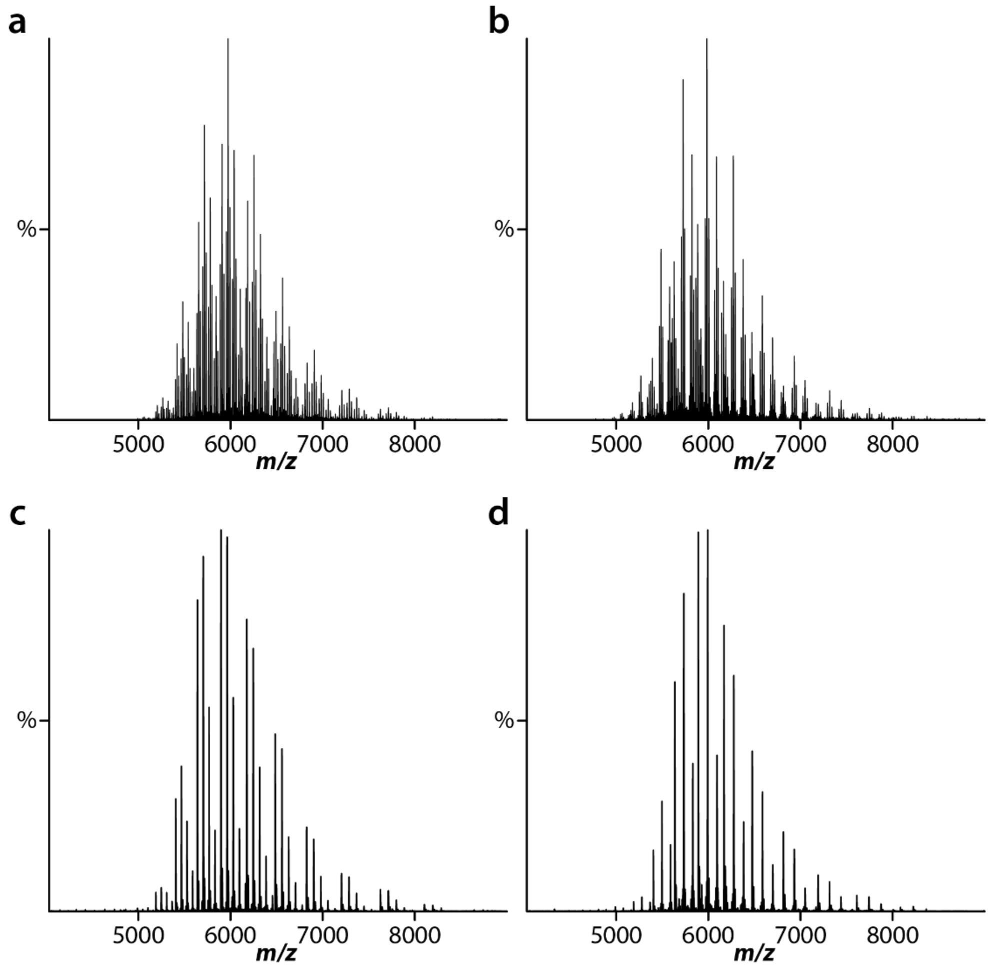

Supplementary Fig. 4. Native mass spectra of ADP and lipid binding to MsbA. The deconvolution of these mass spectra are shown in Figure 2.

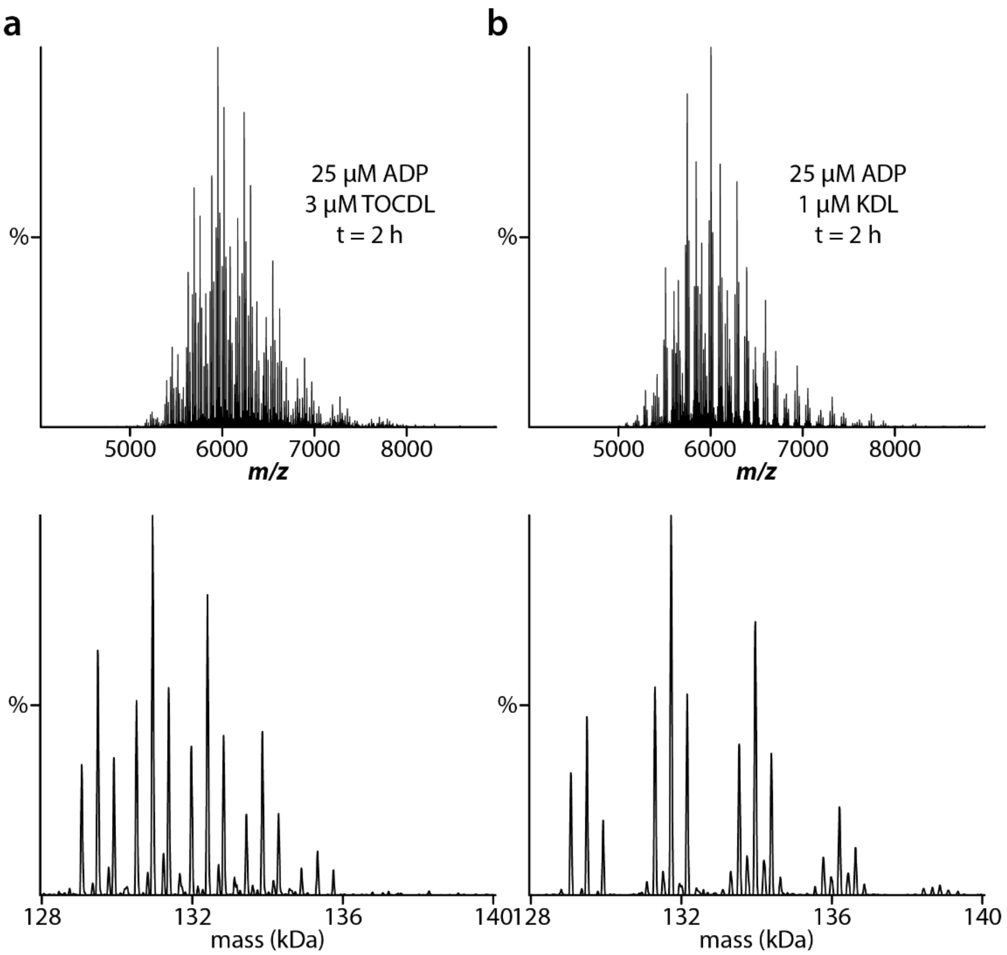

Supplementary Fig. 5. Characterization of ADP and lipid binding to MsbA. Native mass spectra (top) and the deconvolution (bottom) of 0.5 µM MsbA mixed with 10 µM Mg^{2+}, 25 µM ADP and **a** 3 µM TOCDL or **b** 1 µM KDL. Data was recorded after 2-hour incubation.

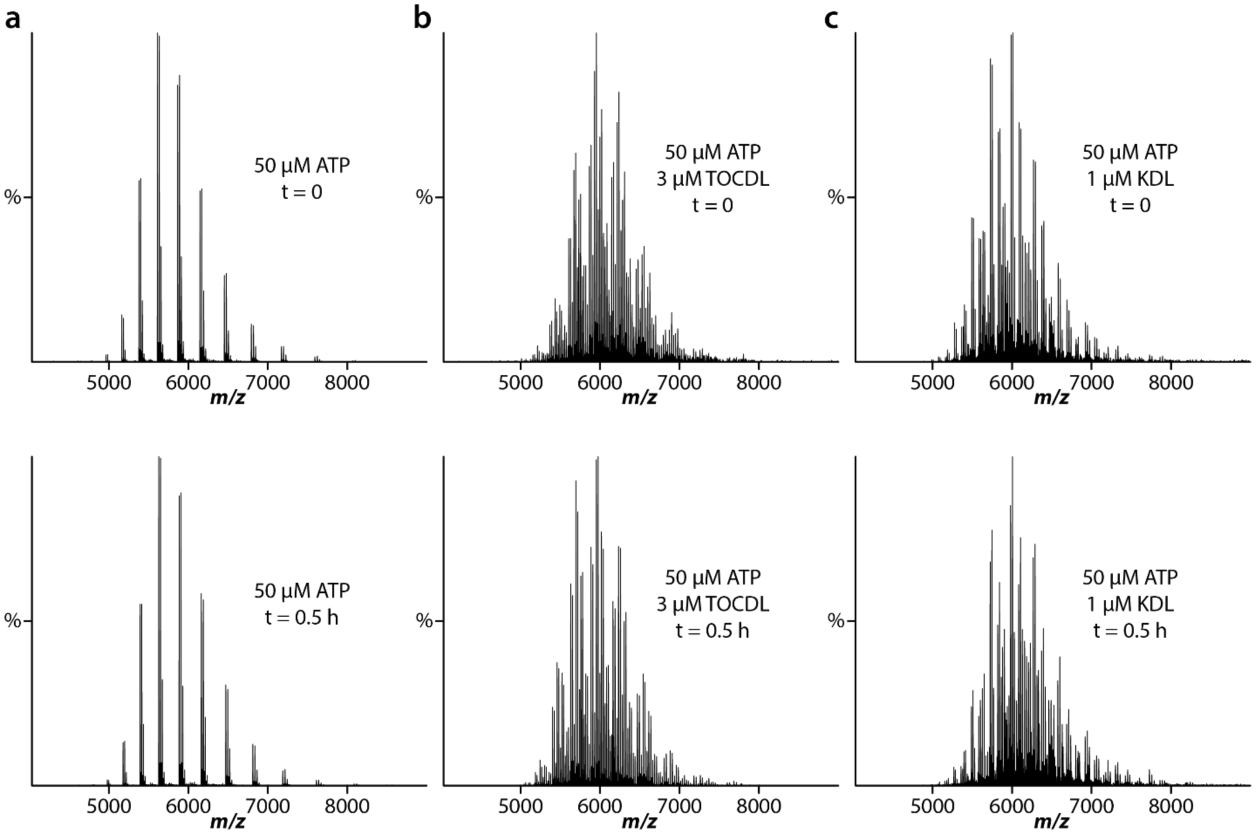

Supplementary Fig. 6. Native mass spectra of ATP and lipid binding to MsbA. The deconvolution of these mass spectra are shown in Figure 3.

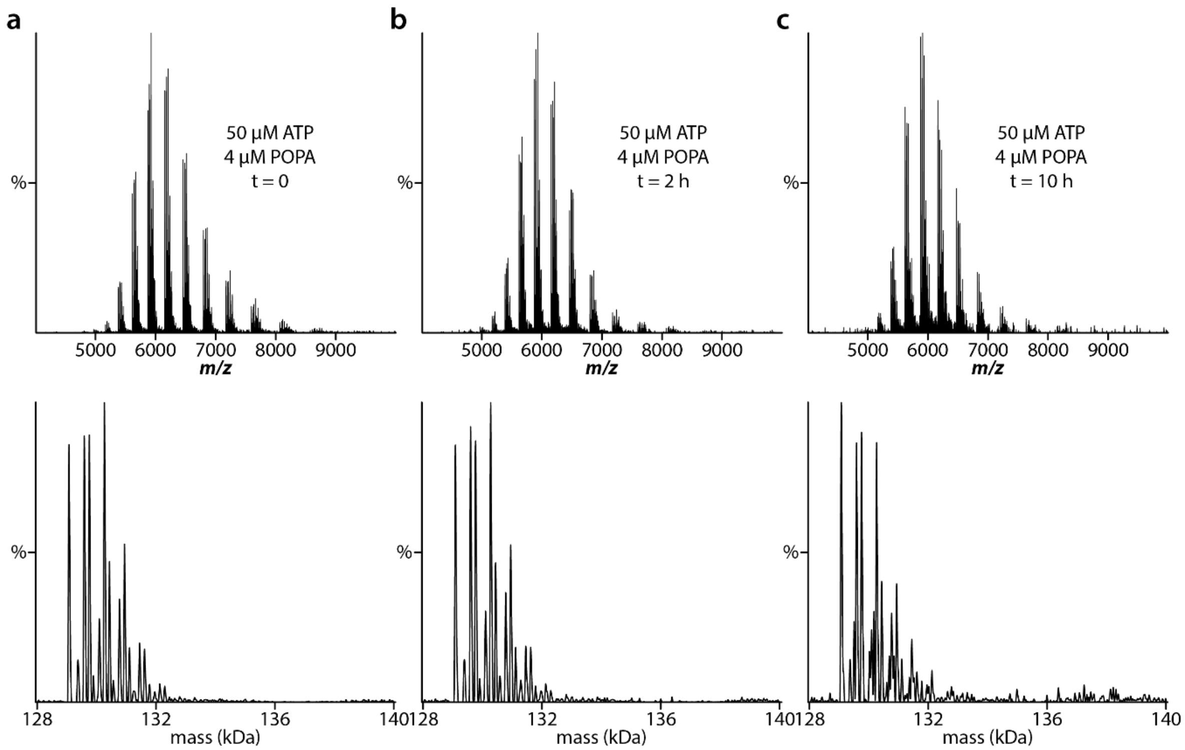

Supplementary Fig. 7. **Native mass spectra of MsbA mixed with ATP and POPA.** Mass spectra (top) and deconvolution (bottom) for 0.5 µM MsbA mixed with 10 µM Mg^{2+}, 50 µM ATP and 4 µM POPA. Data was acquired **a** right after mixing and after incubation for **b** 2 and **c** 10 hours.

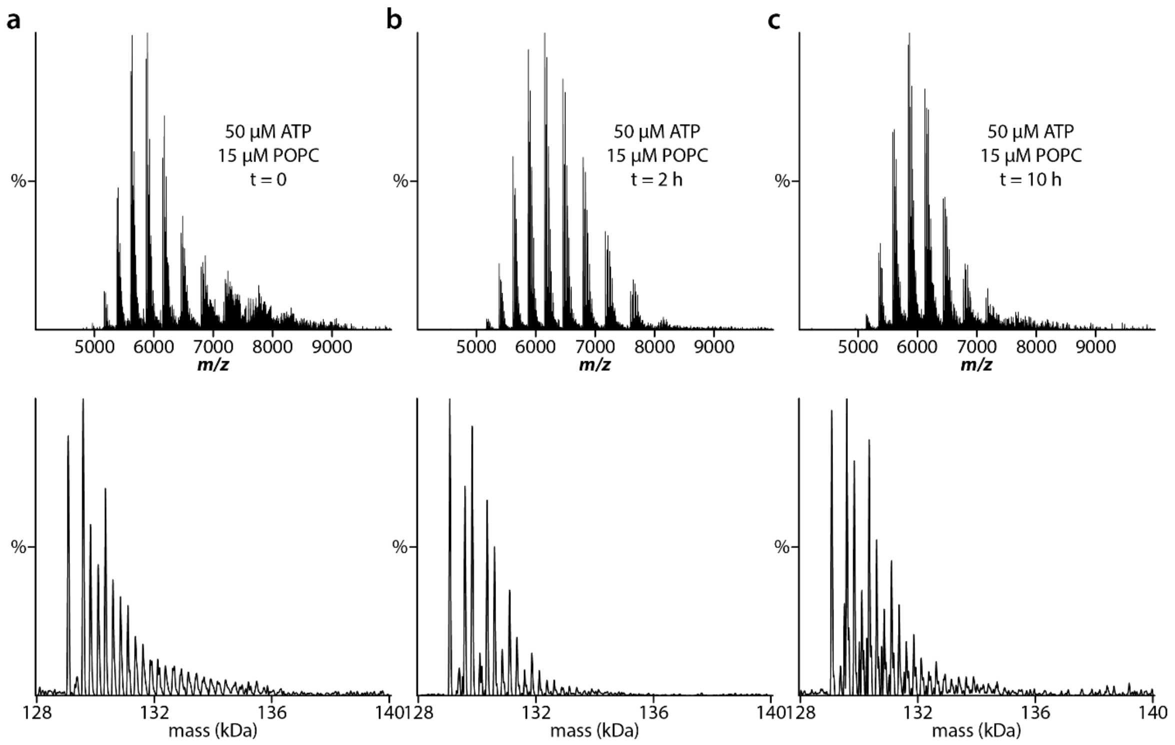

Supplementary Fig. 8. Native mass spectra of MsbA mixed with ATP and POPC. Mass spectra (top) and deconvolution (bottom) for 0.5 µM MsbA mixed with 10 µM Mg^{2+}, 50 µM ATP and 15 µM POPC. Data was acquired **a** right after mixing and after incubation for **b** 2 and **c** 10 hours.

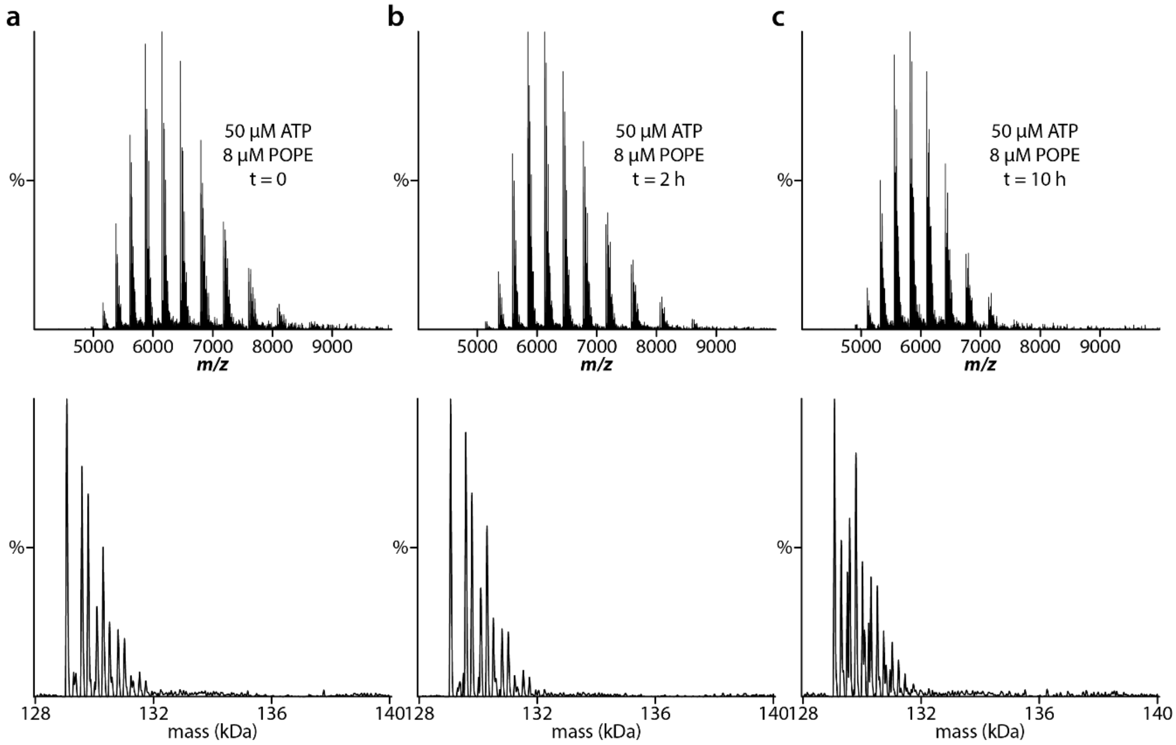

Supplementary Fig. 9. Native mass spectra of MsbA mixed with ATP and POPE. Mass spectra (top) and deconvolution (bottom) for 0.5 µM MsbA mixed with 10 µM Mg^{2+}, 50 µM ATP and 8 µM POPE. Data was acquired **a** right after mixing and after incubation for **b** 2 and **c** 10 hours.

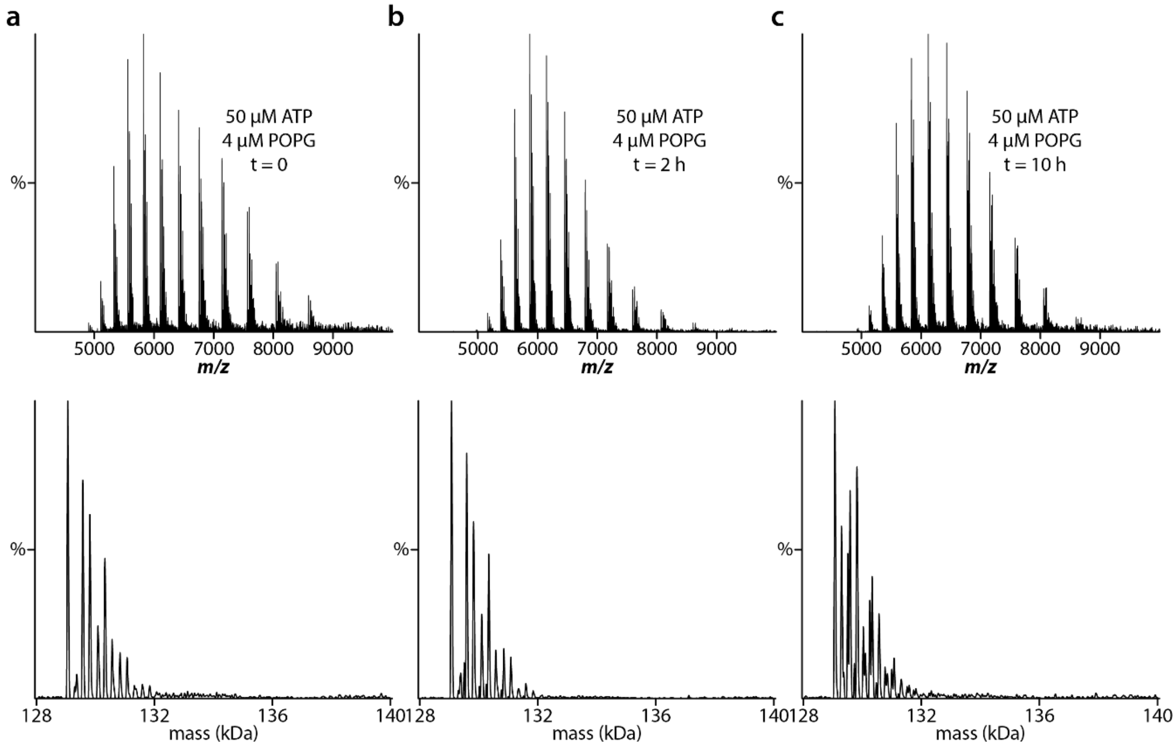

Supplementary Fig. 10. Native mass spectra of MsbA mixed with ATP and POPG. Mass spectra (top) and deconvolution (bottom) for 0.5 µM MsbA mixed with 10 µM Mg^{2+}, 50 µM ATP and 4 µM POPG. Data was acquired **a** right after mixing and after incubation for **b** 2 and **c** 10 hours.

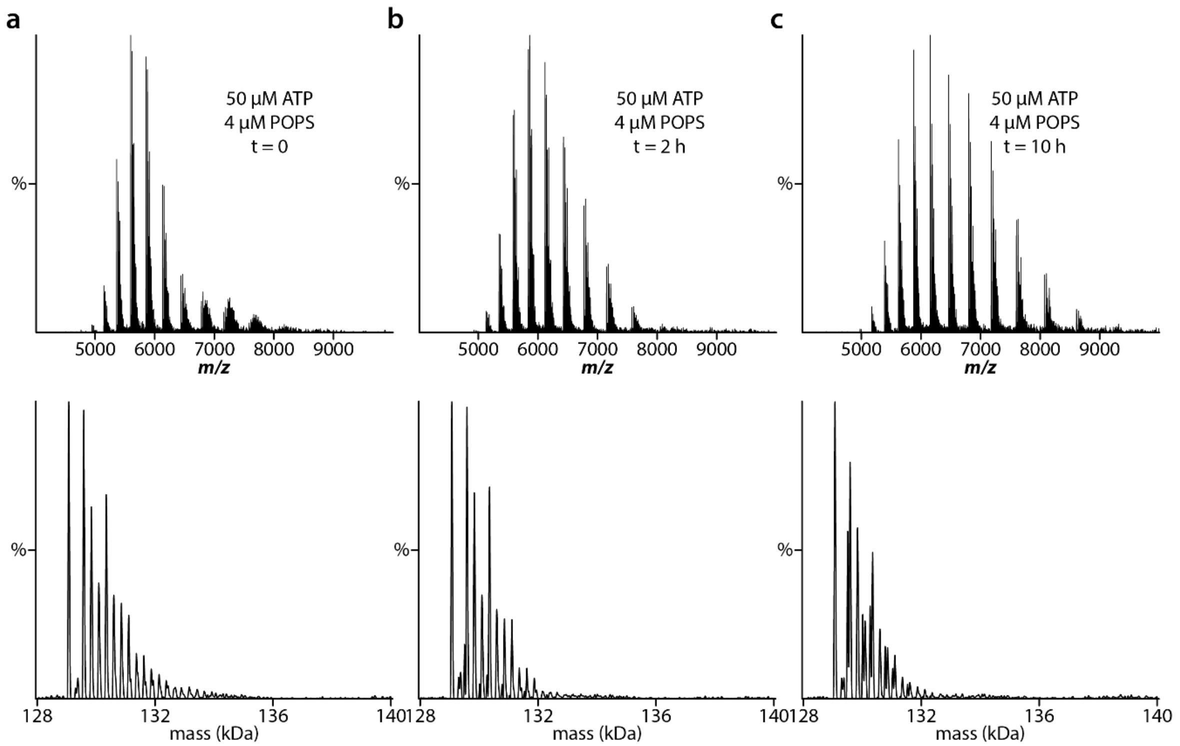

Supplementary Fig. 11. Native mass spectra of MsbA mixed with ATP and POPS. Mass spectra (top) and deconvolution (bottom) for 0.5 µM MsbA mixed with 10 µM Mg^{2+}, 50 µM ATP and 4 µM POPS. Data was acquired **a** right after mixing and after incubation for **b** 2 and **c** 10 hours.

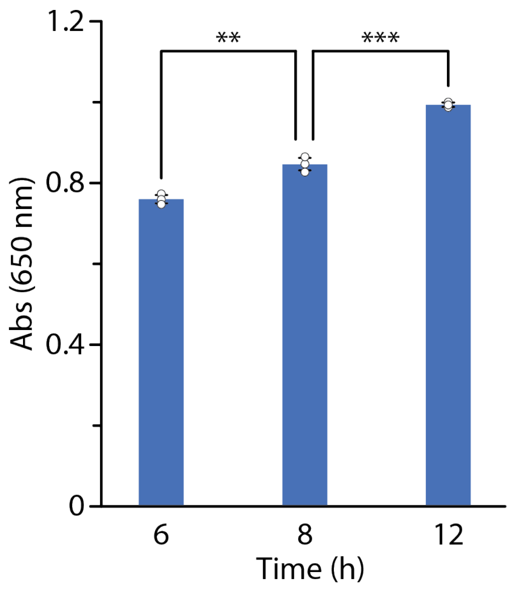

Supplementary Fig. 12. ATPase activity assay of MsbA sample under conditions used for cryoEM. MsbA samples were frozen at a 6-hour time point and the transporter was still active, hydrolyzing ATP. Reported are the mean and standard deviation of the absorbance from a malachite green assay (n = 3, biological replicates). A student's t-test (**$p \leq 0.01$, ***$p \leq 0.001$) was performed to compare different time points. Source data are provided as a Source Data file.

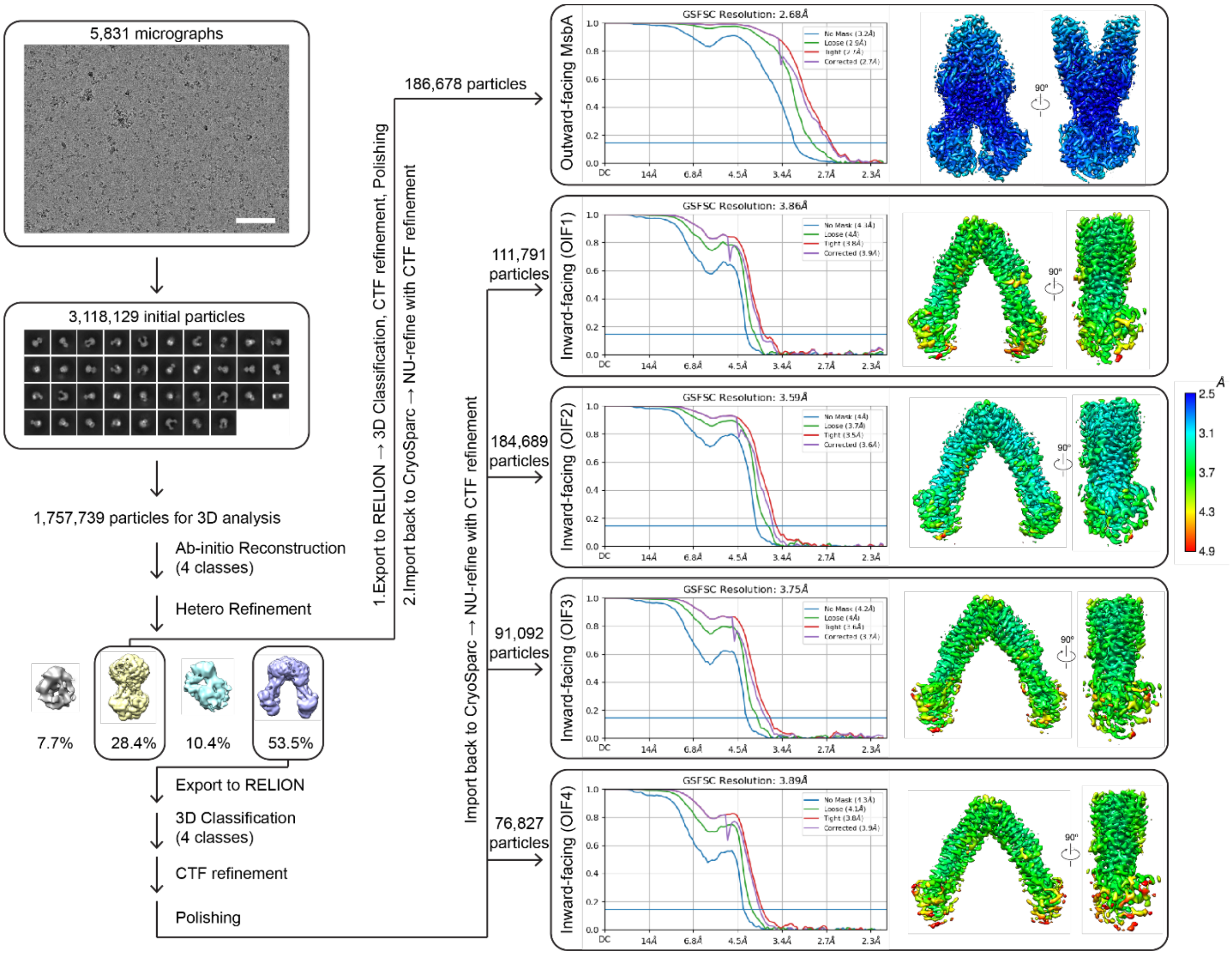

Supplementary Fig. 13. Single-particle cryoEM analysis workflow for MsbA. A representative motion-corrected micrograph is shown along with a 90-nm scale bar. Representative 2D class averages are shown, with the edges of the bounding boxes corresponding to ~273 Å. Fourier shell correlation curves of the final reconstructions are presented. The final sharpened maps, colored with local resolutions, are shown in two distinct orientations. The outward-facing map is contoured using a threshold of 1.4. The inward-facing maps are contoured using a threshold of 0.4. The resolutions reported in this study were determined using the FSC=0.143 criterion.

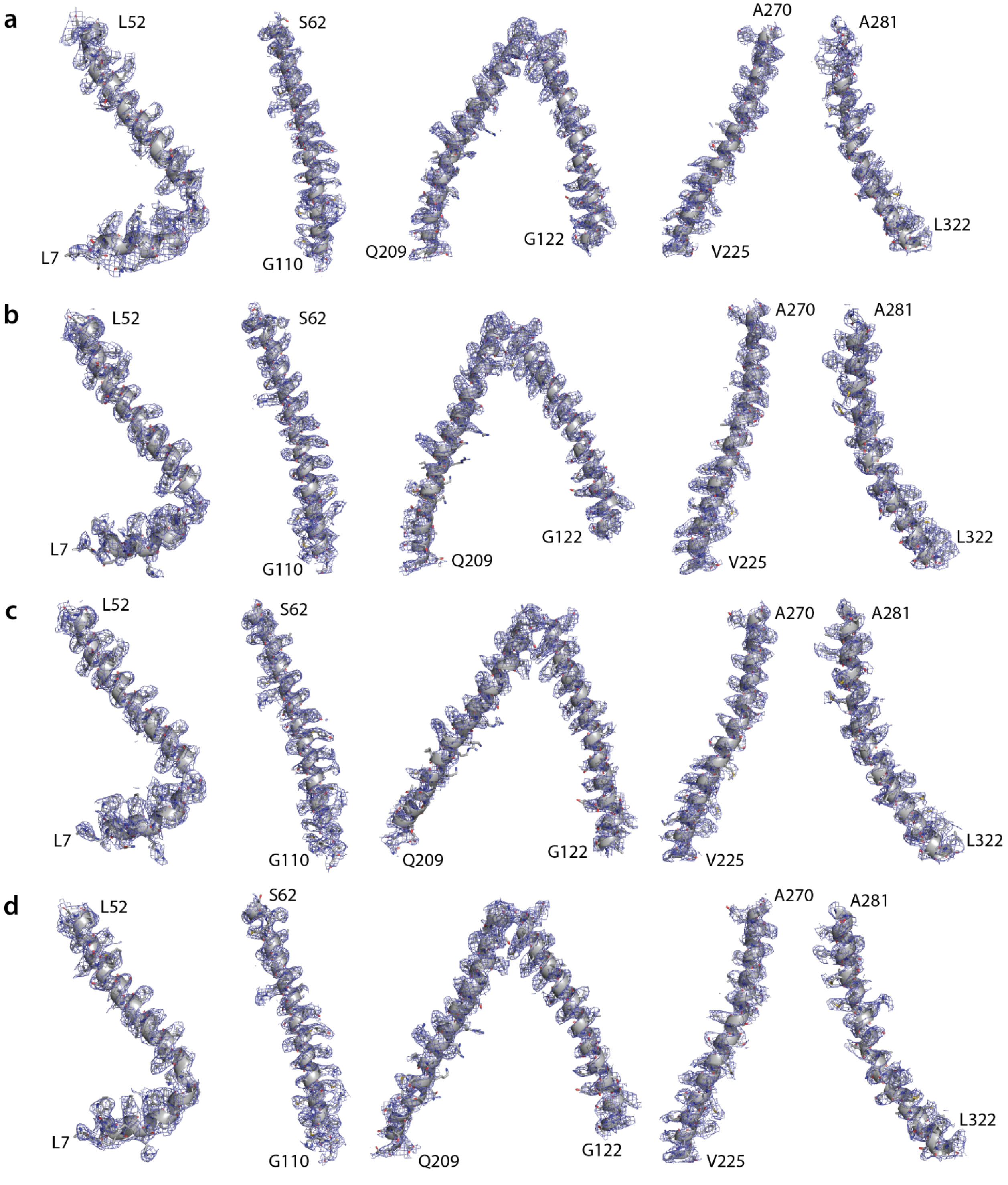

Supplementary Fig. 14. CryoEM density of the inward-facing MsbA structures. Density (contoured at 6 sigma) and atomic model for six transmembrane helices of **a** OIF1, **b** OIF2, **c** OIF3 and **d** OIF4 MsbA structures.

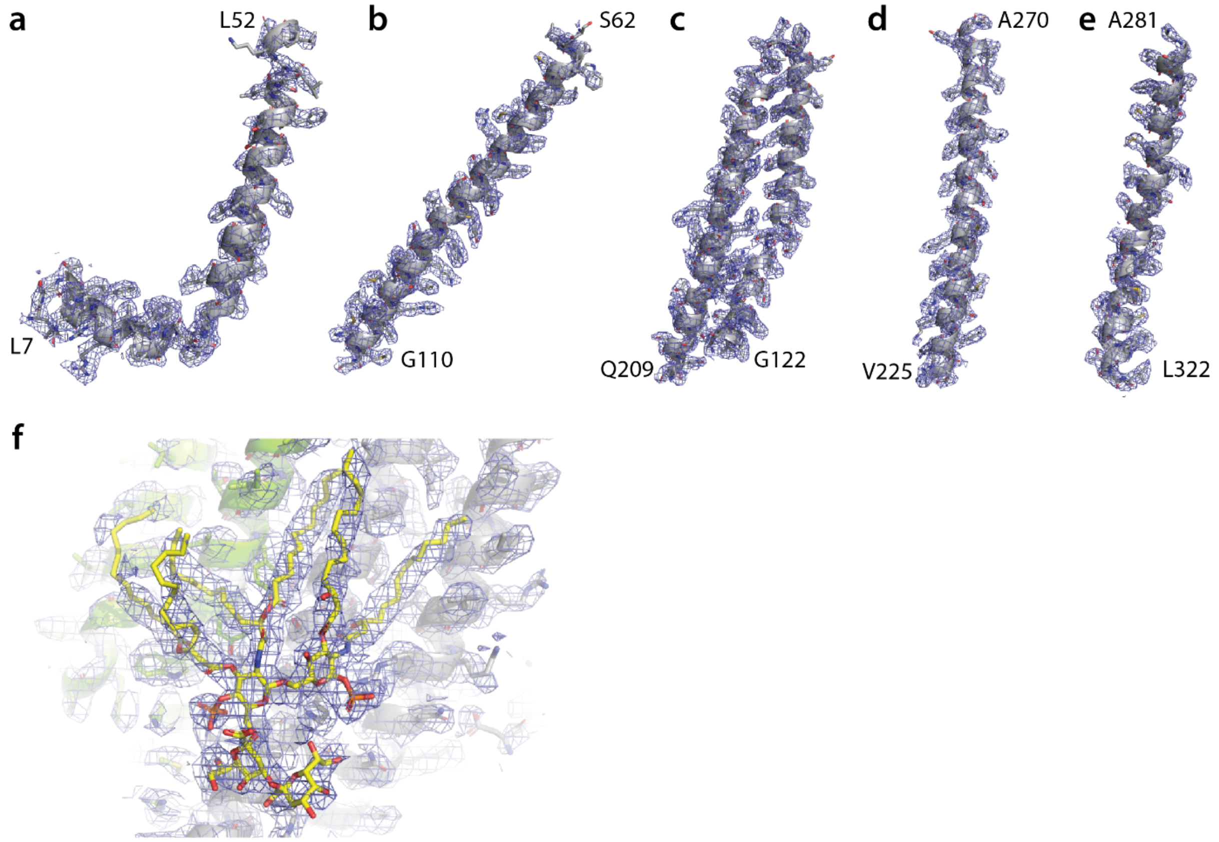

Supplementary Fig. 15. CryoEM density of the open, outward-facing MsbA structure. a-e Density (contoured at 6 sigma) and atomic model for six transmembrane helices. **f** Density (contoured at 6 sigma) for the bound KDL.

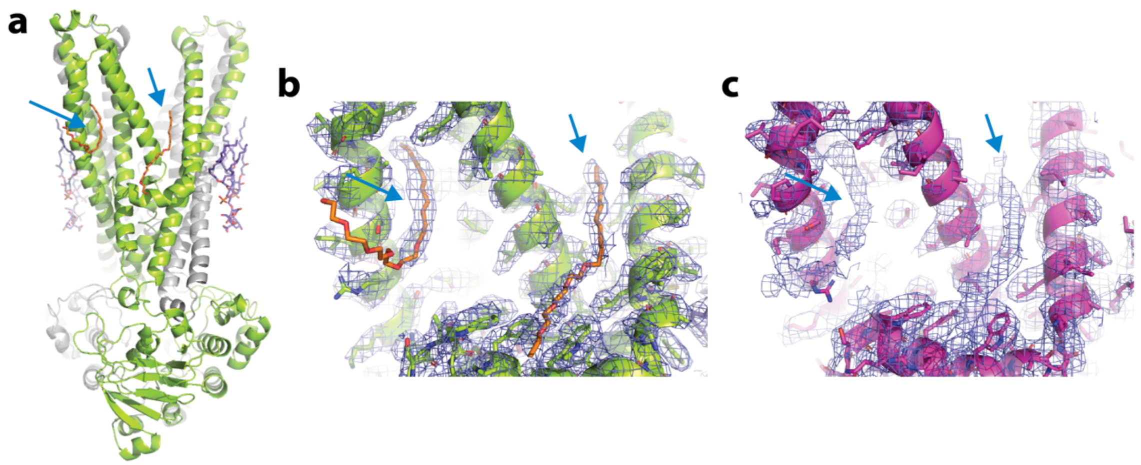

Supplementary Fig. 16. Tube-like density in open, outward-facing MsbA nucleotide-free and bound to ADP and vanadate. a Structure of nucleotide-free MsbA in open, outward-facing conformation shown in cartoon representation. Bound KDL (purple) and $C_{10}E_5$ (orange) are shown in stick representation. The modeled $C_{10}E_5$ detergents are highlighted by a blue arrow. **b** View of the tube-like densities (contoured at 6 sigma) in transmembrane region. The left penetrates a hydrophobic pocket formed by TM5 and TM6. The right is located between TM6 and TM1. **c** Similar view as b but for the open, outward-facing MsbA structure bound to KDL, ADP and vanadate. The density is shown as well (contoured at 4 sigma).

Supplementary Tables

Supplementary Table 1. Theoretical and experimental masses. Reported are the mean and standard deviation of centroid masses.

	Theoretical (Da)	Experimental (Da)
**$MsbA_2$**	129,074	129,067 ± 2
**$MsbA_2(Cu)_2(ATP)_1$**	129,581	129,573 ± 1
**$MsbA_2(Cu)_2(ATP)_2$**	130,088	130,085 ± 2
**$MsbA_2(Cu)_2(ADP)_1$**	129,501	129,494 ± 0
**$MsbA_2(Cu)_2(ADP)_2$**	129,928	129,922 ± 1
**$MsbA_2(Cu)_2(KDL)_1(ATP)_2$**	132,323	132,324 ± 1
**$MsbA_2(Cu)_2(KDL)_2(ATP)_2$**	134,558	134,559 ± 2
**$MsbA_2(Cu)_2(KDL)_3(ATP)_2$**	136,793	136,796 ± 1
**$MsbA_2(Cu)_2(KDL)_4(ATP)_2$**	139,028	139,035 ± 2

Supplementary Table 2. Equilibrium dissociation constants of MsbA-nucleotide interactions determined by native MS. K_D was obtained from fitting a sequential ligand binding model to mole fraction data from titration. Reported are the mean and standard deviation (*n* = 3, biological replicates).

Mg^{2+} (μM)	K_{D1} (ATP, μM)	K_{D2} (ATP, μM)	K_{D1} (ADP, μM)	K_{D2} (ADP, μM)
**10**	47.8 ± 2.5	124.4 ± 6.2	17.8 ± 1.3	62.3 ± 4.7
**50**	49.0 ± 2.1	117.4 ± 6.7	16.7 ± 0.9	63.7 ± 6.6

Supplementary Table 3. Distance between NBDs of open, inward-facing MsbA structures. The reported distance corresponds to the distance between T561 Cα to T561 Cα of the neighboring subunit.

	Distance (Å)
**OIF4 (8TSR)**	91.7
**OIF3 (8TSS)**	89.9
**3B5W**	85.1
**8DMO**	79.3
**OIF2 (8TSQ) and 6BL6**	75.9
**OIF1 (8TSP)**	64.8

Supplementary Table 4. Statistics of cryoEM data collection and processing.

**Microscope**	Krios (University of Chicago)				
**Magnification**	81,000				
**Voltage (kV)**	300				
**Spherical aberration (mm)**	2.7				
**Detector**	K3				
**Camera mode**	Super resolution counting				
**Exposure rate (e^{-}/pixel/s)**	15				
**Total exposure (e^{-}/Å^2)**	50				
**Defocus range (µm)**	-1.0 to -2.5				
**Pixel size (Å)**	0.5325 (1.065 physical)				
**Mode of data collection**	Image shift				
**Energy filter**	20 eV slit				
**Software for data collection**	EPU				
**Number of micrographs**	5,831				
**Symmetry imposed**	C2				
**Box size (pixel)**	256				
**Initial particle images (no.)**	3,118,129				
**Particle images for 3D (no.)**	1,757,739				
	Open, outward-facing MsbA	Open, inward-facing (OIF1)	Open, inward-facing (OIF2)	Open, inward-facing (OIF3)	Open, inward-facing (OIF4)
**Final particle images (no.)**	186,678	111,791	184,689	91,092	76,827
**Map resolution, unmasked (Å)**	3.2	4.3	4.0	4.2	4.3
**Map resolution, masked (Å)**	2.7	3.9	3.6	3.7	3.9
**B-factor used for sharpening (Å^2)**	108.7	168.7	153.1	155.9	151.5
**EMD accession code**	EMD-41596	EMD-41597	EMD-41598	EMD-41560	EMD-41599

Supplementary Table 5. Statistics of cryoEM model refinement and geometry for five MsbA structures.

Model	Open, outward-facing MsbA	OIF1	OIF2	OIF3	OIF4
PDB accession code	8TSO	8TSP	8TSQ	8TSS	8TSR
Composition (#)					
Chains	4	2	2	2	2
Atoms	9298 (Hydrogens: 0)	8888 (Hydrogens: 0)	8918 (Hydrogens: 0)	8918 (Hydrogens: 0)	8888 (Hydrogens: 0)
Residues	Protein: 1146<br>Nucleotide: 0	Protein: 1146<br>Nucleotide: 0	Protein: 1150<br>Nucleotide: 0	Protein: 1150<br>Nucleotide: 0	Protein: 1146<br>Nucleotide: 0
Water	0	0	0	0	0
Ligands	KDL: 2<br>CXE: 4	0	0	0	0
Bonds (RMSD)					
Length (Å) (# > 4σ)	0.004 (0)	0.004 (0)	0.005 (0)	0.004 (0)	0.005 (0)
Angles (°) (# > 4σ)	0.626 (0)	0.752 (2)	0.655 (0)	0.750 (0)	0.832 (0)
MolProbity score	1.34	1.70	1.82	1.91	1.76
Clash score	5.75	10.09	10.06	11.94	11.20
Ramachandran plot (%)					
Outliers	0.00	0.00	0.00	0.09	0.00
Allowed	2.10	2.98	4.28	4.45	3.15
Favored	97.90	97.02	95.72	95.46	96.85
Rotamer outliers (%)	0.00	0.72	0.51	0.41	0.51
Cβ outliers (%)	0.00	0.00	0.00	0.00	0.00
Peptide plane (%)					
Cis proline/general	0.0/0.0	0.0/0.0	0.0/0.0	0.0/0.0	0.0/0.0
Twisted proline/general	0.0/0.0	0.0/0.0	0.0/0.0	0.0/0.0	7.7/0.0
CaBLAM outliers (%)	1.41	1.41	2.71	2.36	1.67
ADP (B-factors)					
Iso/Aniso (#)	9298/0	8888/0	8918/0	8918/0	8888/0
Protein	52.90	40.94	14.99	56.07	59.21
Ligand	47.17	/	/	/	/
Data					
Box					

**Lengths (Å)**	90.53, 75.62, 136.32	67.10, 122.48, 136.32	75.62, 133.13, 135.26	67.10, 135.26, 136.32	67.10, 139.52, 135.26
**Angles (°)**	90.00, 90.00, 90.00	90.00, 90.00, 90.00	90.00, 90.00, 90.00	90.00, 90.00, 90.00	90.00, 90.00, 90.00
**Supplied Resolution (Å)**	2.7	3.9	3.6	3.8	3.9
**Resolution Estimates, Masked (Å)**					
**d model**	3.0	4.2	3.9	4.1	4.2
**d FSC model (0/0.143/0.5)**	2.6/2.6/2.8	3.7/3.8/4.1	3.3/3.5/3.9	3.6/3.7/4.1	3.7/3.8/4.1
**Map min/max/mean**	-6.34/11.07/0.10	-1.73/2.41/0.04	-2.44/3.34/0.04	-1.72/2.30/0.03	-1.53/1.88/0.04
**Model vs. Data**					
**CC (mask)**	0.80	0.74	0.72	0.74	0.76
**CC (box)**	0.64	0.68	0.67	0.67	0.71
**CC (peaks)**	0.63	0.62	0.62	0.60	0.63
**CC (volume)**	0.76	0.71	0.69	0.70	0.72
