# Supplementary material for: Native mass spectrometry and structural studies reveal modulation of MsbA–nucleotide interactions by lipids
Source: Nat Commun. 2024 Jul 15;15:5946. doi: 10.1038/s41467-024-50350-9 (PMC11251056; doi:10.1038/s41467-024-50350-9)
Supplement: Supplementary file 3 — Description of Additional Supplementary Files [file 41467_2024_50350_MOESM3_ESM.pdf]

File name: Supplementary Movie 1
Description: Morph of open, outward-facing MsbA structures in nucleotide-free and ADP and vanadate bound states. The bound KDL is shown in yellow.
